# Clinical application and observation of modified Ivor-Lewis surgery in Siewert type II adenocarcinoma of the Esophagogastric junction

**DOI:** 10.1186/s13019-019-1023-7

**Published:** 2019-11-27

**Authors:** Qifan Yin, Wenhao Wang, Huining Liu, Guang Yang, Shaohui Zhou, Lijun Liu

**Affiliations:** Department of Thoracic Surgery, Hebei General Hospital, 348, West He-Ping Road, Shijiazhuang, 050051 Hebei Province People’s Republic of China

**Keywords:** Adenocarcinoma of the esophagogastric junction (AEG), Siewert type II, Surgical approach

## Abstract

**Background:**

The surgical approach (transthoracic or transabdominal) for patients with Siewert type II adenocarcinoma of the esophagogastric junction (AEG) still remains controversial. We made a bold attempt to adopt the modified Ivor-Lewis surgery, No turning over, thoraco-laparoscopic esophagogastrectomy, two-field lymphadenectomy and intrathoracic anastomosis, to observe the clinical application and effect.

**Method:**

Data of patients with Siewert type II AEG were collected in the Hebei General Hospital from June 2017 to February 2019. The operation time, surgical blood loss, the number of dissected lymph nodes, duration of drainage tube, postoperative complications, the length of postoperative hospital stay were collected to assess the safety and feasibility of modified Ivor-Lewis surgery.

**Results:**

A total of 20 patients with Siewert type II AEG were analyzed in our research, there was no case of turning to thoracotomy, laparotomy or death during the operation.The average operation time, surgical blood loss, amount of dissected lymph nodes, duration of drainage tube, postoperative hospital stay of all enrolled patients was 4.67, 0.57 h, 156, 56.80 ml, 22.55, 3.91, 8.6, 2.21 days, 12.85, 2.5 days respectively. Among all the enrolled patients, one patient(5%) developed anastomotic fistula and one patient(5%) developed hematemesis after operation, eventually, these two patients were discharged successfully.

**Conclusion:**

For patients with Siewert type II AEG, The modified Ivor-Lewis surgery, No turning over, thoraco-laparoscopic esophagogastrectomy, two-field lymphadenectomy and intrathoracic anastomosis, is safe and feasible. The feasibility and safety could be further and better investigated with a RCT to achieve more conclusive results.

## Introduction

In the last two decades, the incidence of adenocarcinoma of the esophagogastric junction (AEG) has been increasing rapidly [1]. Surgical therapy is the only treatment for resectable cases with a curative intent. AEG tumors are commonly classified according to Siewert et al. and divided into three types according to their position referring to the esophagogastric junction, AEG type I to III [2]. Different surgical approaches have been proposed for curative treatment including transhiatally extended gastrectomy (TEG), left or right thoracoabdominal esophagectomy (TAE) with intrathoracic anastomosis [3, 4], and transhiatal esophagectomy [5]. While no major controversies exist about the surgical strategies for AEG type I and III tumors. In the National Comprehensive Cancer Network (NCCN) guideline, Siewert type I/II and type III AEG were included in both the esophageal and gastric cancer guideline [6]. Therefore, it can be assumed that it has been acknowledged that Siewert type I and III tumors can be operated transthoracically as performed for those with esophageal cancers or transabdominally as performed for those with gastric cancers. However, there is still controversy regarding the optimal surgical approach for Siewert type II AEG [7, 8], with some surgeons preferring a transhiatal abdominal approach while others favor a thoracic approach. Some studies have shown that a thoracoabdominal approach is needed to achieve sufficient mediastinal and abdominal lymphadenectomy as well as negative resection margins [9]. On the other hand, there are indications for higher morbidity rates after thoracoabdominal surgery and poor outcome of patients even after resection and radical lymphadenectomy [10]. In recent years, Traditional open Ivor-Lewis surgery has been proficiently performed to cure AEG in our departement, which has some shortcomings such as needing of turning over intraoperation, longer operative time, larger surgical trauma, more postoperative complications, longer hospital stay, more severe postoperative pain.In order to shorten operative time and hospital stay, relieve surgical trauma and postoperative pain. Therefore, We made a bold attempt to adopt the modified Ivor-Lewis surgery, No turning over, thoraco-laparoscopic esophagogastrectomy, two-field lymphadenectomy and intrathoracic anastomosis, to observe the clinical application and effect.

## Material and method

### Patients

20 patients with Siewert type II AEG were enrolled into our research from June 2017 to February 2019, all of whom had underwent modified Ivor-Lewis Surgery:No turning over, thoraco-laparoscopic esophagogastrectomy, two-field lymphadenectomy and intrathoracic anastomosis, in our department in Hebei General Hospital. 14 male and 6 female, age from 52 to 74, average age 64.5, 4.7. 10 patients had a history of smoking;The average BMI of enrolled patients was 24.12 ± 2.70 kg/m^2^; As for comorbidities, 7 patients with hypertension,6 patients with type 2 diabetes disease, and 3 patients with coronary heart disease. The characteristics of the enrolled patients were shown in Table 1.

**Table. 1:** The patients characteristics

Patients’ characteristics		Number of patients(*n* = 20)
Age	Average	64.5 ± 4.7
	Range	52—74
Gender	Male	14
	Female	6
Smoking status	Smoker	10
	Non-smoker	10
BMI		24.12 ± 2.70 kg/m^2^
Comorbidity		
Hypertension	Present	7
	Absent	13
T2DM	Present	6
	Absent	14
Coronary heart disease	Present	3
	Absent	17

The inclusion criteria were as follows criteria: 1. Studied patients with AEG were pathological examination confirmed;2. The staging was limited to IIA or IIB according to TNM classification of gastric cancer as described in the 7th edition of the American Joint Committee on Cancer (AJCC);3. Siewert type II AEG; 4.The lesion length is less than 4 cm;5.The KPS score ≥ 90 points.

The exclusion criteria were; 1.The staging was III or IV according to TNM classification;2. Siewert type I, III AEG;3.Neoadjuvant chemo−/ chemoradio-therapy before surgery;4.The KPS score, 90 points;5.Previous history of chest or abdominal surgery;6.Patients with sereve cardiac, pulmonary, brain, renal complications cannot tolerate surgery.

The study was approved by the local Ethical Committee of the Hebei General Hospital and was conducted in accordance with the ethical principles of the Declaration of Helsinki. All patients provided written informed consent.

### Surgical procedure

1. Single lumen endotracheal tube was intubated after successfully anesthesia, Patients in the study were placed in the left supine position at 45 degrees, the upper abdomen and the right chest were disinfected and drapped at the same time after properly fixed.
2. Transabdominal surgery: Tilt the operating bed 45 degrees to the right, so that the patient is supine, mobilization of stomach and the lower esophagus, division of the diaphragm and abdominal lymphadenectomy was performed in the video-assisted laparoscopy.The lower esophagus was dissected by linear cut stapler. Make a 5 cm vertical abdominal incision in the upper epigastrium, take out the stomach and resect the tumor and than make a gastric tube along the greater curvature by linear cut staplers, oversuture the incision edge. After mobilization of the stomach, division of the left gastric artery at its origin, the lymphadenectomy along the celiac axis and suprapancreatic region was performed (according to a D2-lymphadenectomy for gastric carcinoma). The abdomen was closed.
3. Transthoracic surgery: Tilt the operating bed 45 degrees to the left, so that the patients is in lateral position. Mobilization of the esophagus, thoracic lymphadenectomy and intrathoracic anastomosis in the video-assisted thoracoscopy. The gastric tube was pulled into the thorax through the hiatus. The reconstruction of the digestive tract was performed with an esophagogastrostomy (side-to-side) in the tip of the right pleural cavity by a linear cut stapler. The closure of the gastric tube and esophagus was achieved by a linear cut stapler. By oversuturing the anastomosis and the blind end of the esophagus and gastric tube, a functional anastomosis was created. The chest was drained with one tube parallel to the gastric tube in the posterior mediastinum with its superior tip at the anastomosis and one tube in the recessus of the diaphragm.The thorax was closed.

### Postoperative data collection

The operation time, surgical blood loss, the number of dissected lymph nodes, duration of drainage tube, postoperative complications, the length of postoperative hospital stay were collected by the third author.

## Result

All the enrolled patients successfully completed surgery, there was no case of turning to thoracotomy, laparotomy or death during the operation.Data collection and statistical analysis were performed on all enrolled patients. All quantitative data are expressed as mean, standard deviation (SD).The postoperative data of the enrolled patients were shown in Table 2.

**Table. 2:** Postoperative data collection

Postoperative observed indicators		Data
Operation time	Average	4.67 ± 0.57 h
	Range	3.4—5.5 h
Surgical blood loss	Average	156 ± 56.80 ml
	Range	60—250 ml
The number of dissected lymph nodes	Average Range	22.55 ± 3.91 16—31
Duration of drainage tube	Average	8.6 ± 2.21 days
	Range	6—13 days
The length of postoperative hospital stay	Average Range	12.85 ± 2.5 days 9—17 days

- Operation time; The average operation time of all included subjects was 4.67, 0.57 h, the minimum was 3.4 h and the maximum is 5.5 h.
- Surgical blood loss; The average surgical blood loss of all enrolled patients was 156, 56.80 ml, the minimum amount of blood loss was 60 ml, the maximum amount of blood loss was 250 ml.
- The number of dissected lymph nodes; In all enrolled patients, the average amount of dissected lymph nodes was 22.55 ± 3.91, the minimum was 16 and the maximum was 31.
- Duration of drainage tube; The average duration of drainage tube of all enrolled patients was 8.6, 2.21 days, the minimum duration of drainage tube was 6 days, the maximum duration was 13 days.
- The length of postoperative hospital stay; the mean postoperative hospital stay was 12.85 ± 2.5 days, the minimum postoperative hospital stay was 9 days, the maximum postoperative hospital stay was 17 days.
- Postoperative complications; Among all the enrolled patients, one patient(5%) developed anastomotic fistula on the third day after surgery. Postoperative hematemesis occurred in one patient in the seventh day after operation, eventually, these two patients were discharged successfully. No other serious complications occurred in the remaining patients. Perioperative complications were shown in Table 3.

**Table. 3:** Analysis of patients’ complications

Complications	N = patients(%)
Anastomotic fistula	1(5%)
Postoperative hematemasis	1(5%)
Switch to laparotomy	0(0%)
Switch to thoracotomy	0(0%)
Perioperative death	0(0%)

## Discussion

After decades of research progress, the operation methods of AEG include transthoracic approach, transabdominal approach and combined thoracoabdominal approach. However, different surgical methods still have different advantages and disadvantages. According to NCCN guideline, the therapeutics of Siewert type I/II AEG has been classified as to that of esophageal carcinoma, the transthoracic approach is considered as the recommended surgical procedure [6]. Siewert type III AEG tumors are to be considered as upper gastric cancers and the surgical treatment should be performed transabdominally total gastrectomy alongside with D2 lymphadenectomy [11]. However, based from the results obtained from several Asian studies and according to the Japanese gastric cancer treatment guideline [1, 6, 7, 11–13], the biological behavior and pathological features of Siewert type II and III AEG in Asian patients bear more resemblance to gastric cancer than that of esophageal cancer. From the current studies [14], the treatment of Siewert type II AEG, in regard to the selection of surgical approach and lymph node dissection, is still a controversial dilemma and is worthy for further exploration. Previous literatures have shown that the transthoracic and transabdominal approach were the most common surgical approaches for Siewert type II AEG and their benefits were also compared [6, 11, 12, 14].The operation method of Ivor-Lewis selects the advantages of transthoracic or transabdominal approach operation for Siewert type II AEG, enabling to fully mobilize esophagus and stomach, ensuring no residual cancer cells in incisal margin, completely dissecting lymph nodes, achieving radical treatment. However, The results showed that the thoraco-abdominal approach not only failed to improve the long-term survival of the patients, but also increased the perioperative complications and mortality [4, 10].The main reason is because the traditional open Ivor-Lewis surgery has longer operative time, larger surgical trauma, more postoperative complications.

Compared to traditional open Ivor-Lewis surgery, The modified Ivor-Lewis surgery, No turning over, thoraco-laparoscopic esophagogastrectomy, two-field lymphadenectomy and intrathoracic anastomosis, carried out in our department has advantages in the following aspects. First, all enrolled patients were placed in the left supine position at 45 degrees before the operation, we changed the position of the patients by adjusting the angle of the operating bed during the operation, avoiding the process of intraoperative turning over and disinfection, shortening the unnecessary operative time. Second, the modified Ivor-Lewis surgery is able to mobilize longer esophagus and make a longer gastric tube, achieve thoracic and abdominal lymphadenectomy through the combination between thoracoscopy and laparoscopy. These ensure no residual cancer cells on the stump, realize the goal of radical cure. Third, the combination between thoracoscopy and laparoscopy can relieve surgical trauma and postoperative pain, accelerate postoperative rehabilitation, reduce postoperative complications compared to traditional thoracotomy and laparotomy.

Some limitations have to addressed in our study. First, the enrolled patients were limited, which inevitably contributed to some bias. Second, It was a challenge for operator and assistant that the patient’s position couldn’t completely meet conventional anatomical position.

## Conclusion

In conclusion, Compared to traditional open Ivor-Lewis surgery, The modified Ivor-Lewis surgery, No turning over, thoraco-laparoscopic esophagogastrectomy, two-field lymphadenectomy and intrathoracic anastomosis, is safe and feasible. The feasibility and safety could be further and better investigated with a RCT to achieve more conclusive results.

## Abbreviations

AEG: adenocarcinoma of the esophagogastric junction
AJCC: American Joint Committee on Cancer
NCCN: National Comprehensive Cancer Network
SD: standard deviation
TAE: thoracoabdominal esophagectomy
TEG: transhiatally extended gastrectomy
